# Advancements in Brain Aneurysm Management: Integrating Neuroanatomy, Physiopathology, and Neurosurgical Techniques

**DOI:** 10.3390/medicina60111820

**Published:** 2024-11-06

**Authors:** Ligia Gabriela Tataranu, Octavian Munteanu, Amira Kamel, Karina Lidia Gheorghita, Radu Eugen Rizea

**Affiliations:** 1 Neurosurgical Department, Carol Davila University of Medicine and Pharmacy, 020022 Bucharest, Romania; ligia.tataranu@umfcd.ro (L.G.T.); rizea.radu.eugen@gmail.com (R.E.R.); 2 Neurosurgical Department, Bagdasar-Arseni Clinical Emergency Hospital, 041915 Bucharest, Romania; kamel.amyra@yahoo.com; 3 Anatomy Department, Carol Davila University of Medicine and Pharmacy, 020022 Bucharest, Romania; 4 University Emergency Hospital, 050098 Bucharest, Romania; 5 Pediatric Neurology Private Practice, 031871 Bucharest, Romania; karina.gheorghita@gmail.com

**Keywords:** brain aneurysms, neuroanatomy, neurosurgery, vascular structures, pathophysiology, microsurgical clipping, endovascular coiling, flow diversion techniques, preoperative planning, postoperative care, multidisciplinary approach, intraoperative navigation

## Abstract

Brain aneurysms, characterized by abnormal bulging in blood vessels, pose significant risks if ruptured, necessitating precise neuroanatomical knowledge and advanced neurosurgical techniques for effective management. This article delves into the intricate neuroanatomy relevant to brain aneurysms, including the vascular structures and critical regions involved. It provides a comprehensive overview of the pathophysiology of aneurysm formation and progression. The discussion extends to modern neurosurgical approaches for treating brain aneurysms, such as microsurgical clipping, endovascular coiling, and flow diversion techniques. Emphasis is placed on preoperative planning, intraoperative navigation, and postoperative care, highlighting the importance of a multidisciplinary approach. By integrating neuroanatomical insights with cutting-edge surgical practices, this article aims to enhance the understanding and treatment outcomes of brain aneurysms.

## 1. Introduction

**Figure 1:**
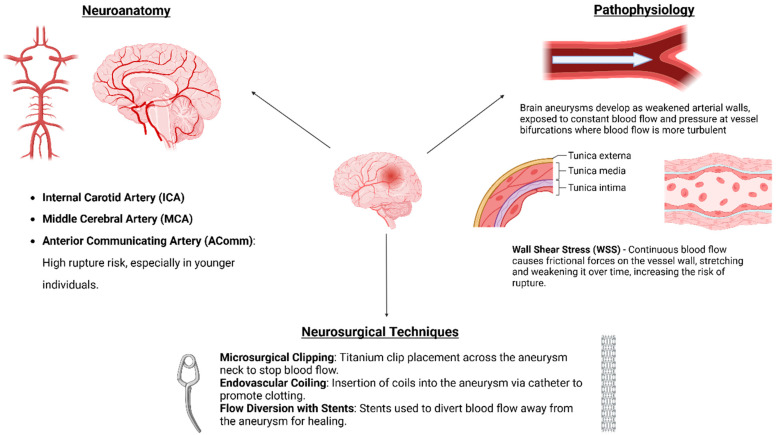
A summary figure illustrating key points about aneurysm anatomy, pathophysiology, and neurosurgical treatment.

Intracranial aneurysm (IA) is a cerebrovascular disorder characterized by an abnormal focal dilation caused by a weakened region within the wall of a cerebral artery. Both microsurgical and endovascular treatments aim to exclude aneurysms from cerebral circulation, thereby preventing their rupture. Despite significant advancements in endovascular techniques, achieving complete and durable aneurysm occlusion remains a complex challenge. The underlying biological mechanisms driving aneurysm growth and recanalization are not yet fully elucidated. An aneurysm represents a weakened area within a blood vessel wall that protrudes, disrupting normal blood flow and potentially leading to a hemorrhagic stroke. Vascular microsurgery seeks to repair aneurysms by isolating them from the arterial circulation while avoiding rupture or damage to adjacent tissues [1,2,3]. This surgical procedure involves the careful dissection of surrounding structures and the retraction of brain tissue to expose the aneurysm neck, where a titanium clip is applied to prevent rupture. Errors in this procedure can result in ischemic injury or hemorrhage [4,5,6]. In cases of large aneurysms with complex anatomical features, multiple clips may be necessary. Given the high risks associated with these procedures, there is limited opportunity for residents to practice, underscoring the importance of neurosimulation tools. These tools enhance training by providing opportunities to gain experience and manage errors within a controlled, safe environment [7,8]. Training methods include biological tissue models [9], mannequins [10], and virtual reality (VR) systems [11]. Prominent VR systems include NeuroTouch, designed for tumor removal, and ImmersiveTouch, which focuses on vascular neurosurgery. However, many VR systems designed for vascular applications fail to adequately model brain tissue [12,13] (Figure 1).

Research has demonstrated significant sex differences in the prevalence of IA. A recent study reported that incidental unruptured intracranial aneurysms (UIAs) are notably more prevalent in females than in males, supported by an analysis of over 14,000 adults, which revealed a higher odds ratio (OR) for females (OR, 1.92 [95% CI, 1.33–2.84]) [14]. Long-term studies and meta-analyses further indicate that females are at a higher risk of developing de novo aneurysms, with ORs ranging from 1.81 to 3.83 [15,16].

Additionally, female gender has been identified as an independent risk factor for the growth of unruptured cerebral aneurysms. A long-term follow-up study involving 87 patients found that females were more likely to experience aneurysm growth of at least 1 mm after adjusting for age (OR, 3.36 [95% CI, 1.11–10.22]) [17]. Another study analyzing 1325 unruptured aneurysms concluded that female sex was the only significant risk factor for aneurysm growth (*p* = 0.0281) [18].

The location of aneurysms also varies by sex. An analysis of 682 aneurysms revealed that females are more likely to have aneurysms along the internal carotid artery (54% in females vs. 38% in males), whereas males more commonly present with aneurysms along the anterior cerebral artery (29% in males vs. 15% in females; *p* = 0.001) [19]. These findings were corroborated by another study of 444 aneurysms, which showed a higher incidence of anterior cerebral artery aneurysms in males (81% vs. 49% in females, *p* < 0.0001) and a greater prevalence of internal carotid artery aneurysms in females (64% vs. 24% in males, *p* < 0.0001) [20].

A recent study involving 1277 patients with ruptured IA identified female sex as a significant risk factor for the presence of multiple aneurysms (relative risk ratio, 1.80 [95% CI, 1.31–2.48]) [21]. The underlying causes of these sex differences in aneurysm location and multiplicity may involve hormonal influences on vascular remodeling and a higher propensity for vessel wall weakness in females [20]. Additionally, hemodynamic factors, such as larger vessel diameters in males and higher wall shear stress in certain arteries in females, may contribute to the observed sex differences in aneurysm prevalence and location [22].

## 2. Overview of Cerebral Vasculature

In the human vascular system, blood flow can be classified as either laminar or turbulent, depending on its velocity and the geometric characteristics of the vessels. Laminar flow is typically observed in large, straight vessels, while turbulent flow is more common in smaller, curved vessels. Under turbulent flow conditions, the blood velocity is relatively high, and the geometric complexity of the vessels increases, which in turn exerts mechanical forces on the vessel walls. Persistent abnormal blood flow can disrupt endothelial cell function, making it a key factor in the development of IA [23,24].

The mechanical forces exerted by blood flow on vessel walls include endothelial cell stretch, the impulse force on the vessel wall, and the tangential force, commonly referred to as wall shear stress (WSS). WSS is measured in Pascals (Pa) or Newtons per square meter (N/m^2^) and represents the friction between layers of blood moving at different velocities within the vessel. In healthy large blood vessels, the average WSS is approximately 15 dynes/cm^2^, although it varies considerably across different regions of the vascular system due to the complex vessel architecture. For instance, WSS ranges from 9.5 to 15 dynes/cm^2^ in the common carotid artery and from 3.9 to 4.9 dynes/cm^2^ in the femoral artery. In vessels with large diameters and regular shapes, WSS tends to remain stable; however, in curved or bifurcated vessels, abnormal WSS can continuously stimulate the vessel walls, leading to compromised structural integrity and triggering inflammatory responses [25,26].

The brain, which receives 20% of the body’s circulating blood volume, is characterized by a particularly intricate vascular anatomy, especially within the circle of Willis, which contains numerous curves and bifurcations [27]. Computational flow dynamics (CFD) is a widely utilized tool for investigating and simulating hemodynamic conditions in cerebral arteries. CFD allows for the visualization and measurement of vessel dynamics, including WSS and the oscillating shear index (OSI), the latter of which reflects the resilience of vessel walls [28]. This technology enables non-invasive assessment of blood dynamics, facilitating the calculation of blood flow quantity and distribution across different regions of the brain [29].

Hypertension, smoking, alcohol consumption, environmental factors, and genetic predispositions have all been identified as significant risk factors for the development of IA (IAs). Research has demonstrated that abnormal hemodynamics is closely associated with the occurrence and progression of IAs [24,30]. The circle of Willis is the most common site for IAs due to its unique anatomical structure, which is particularly susceptible to irregular hemodynamic stimulation. According to a study, approximately 90% of saccular IAs are located within the circle of Willis, with the remainder predominantly found at vessel bifurcations [31]. This circle serves as a crucial anastomotic arterial ring, connecting the anterior and posterior circulations as well as the left and right hemispheres. A relevant study reported that in autopsy examinations, the integrity of the circle of Willis is observed in only 40% of the population, with anatomical variations present in more than half of individuals, resulting in diverse and complex intracranial blood flow patterns [32].

In addition to congenital variations in the circle of Willis, many individuals experience vascular tissue abnormalities due to genetic factors, which increase their susceptibility to aneurysm formation under hemodynamic stress. The elastin layer in the vascular wall of IA is often absent, potentially due to abnormal gene expression of structural proteins. For instance, between 2% and 10% of patients with polycystic kidney disease develop aneurysms because of a genetic inability to encode a specific vascular structural protein. Similarly, in Marfan syndrome, IA can arise due to the lack of collagen-encoding genes [33,34]. Ehlers–Danlos syndrome is another genetic disorder that can contribute to the development of IA by affecting connective tissues [35]. Both Marfan and Ehlers–Danlos syndromes can also predispose individuals to a range of other conditions, including dermatological issues, cardiovascular diseases, gastrointestinal disorders, osteoarthritis, and even organ ruptures [36,37].

To better understand the factors influencing aneurysm location within cerebral arteries, researchers have explored the relationship between aneurysm location and wall shear stress (WSS) levels. High WSS and high WSS gradient environments have been shown to induce destructive remodeling of the arterial wall, similar to the processes involved in aneurysm initiation. Persistent primitive olfactory arteries (PPOA) and azygos pericallosal arteries, although rare, have been identified as potential sites for aneurysm formation due to their distinctive structural characteristics, highlighting the significance of anatomical variations in aneurysm development. Furthermore, increased regional blood flow under high WSS conditions has been implicated in the formation of nascent aneurysms, which exhibit histological features akin to those observed in human aneurysms, including the absence of the internal elastic lamina and a thinned media layer [38,39].

In the progression of aneurysms, longitudinal blood flow impinging on the vessel wall is recognized as a critical risk factor for aneurysm growth, as continuous blood flow exacerbates damage to the vascular structure. Low wall shear stress (WSS) combined with a high oscillatory shear index (OSI) has been shown to promote aneurysm growth, with regions of growth often exhibiting low shear conditions and increased oscillatory flow patterns. This low WSS environment is analogous to the conditions that favor the formation of atherosclerotic plaques, which may explain why atherosclerotic lesions are frequently observed within aneurysms. Low WSS also fosters vascular inflammation and endothelial dysfunction, which gradually compromise the structural integrity of the vascular wall, thereby facilitating aneurysm development. While high WSS is typically associated with the initiation of aneurysms, low WSS is more closely linked to the rupture of aneurysms. Although the precise relationship between WSS levels and aneurysm growth or rupture is still a subject of debate, there is a general consensus that both low and high WSS play a significant role in influencing the natural history of aneurysms [40,41].

## 3. Anatomical Considerations

IA can be classified through various schemes that address different aspects of the condition. IA represent a heterogeneous group of lesions, with the most prevalent type being saccular or berry aneurysms, which account for approximately 90% of all aneurysms. Other types include fusiform aneurysms, which involve an extended segment of the vessel; traumatic aneurysms; mycotic aneurysms, which are associated with underlying infectious processes; dissecting aneurysms; and microaneurysms, typically found on small perforator vessels as a result of chronic hypertension. Around 85% of aneurysms are located in the anterior circulation of the circle of Willis [42]. Clinically, they are categorized based on their rupture status: either ruptured or unruptured. Morphologically, aneurysms are divided into saccular and non-saccular types, with non-saccular IAs further subdivided into fusiform, dolichoectatic, and dissecting aneurysms. In terms of their location within the intracranial circulation, aneurysms are classified as either anterior circulation or posterior circulation. The angioarchitecture of aneurysms is particularly important for planning management strategies and is categorized based on neck size and its relationship to the dome. These classification systems not only provide a detailed description of aneurysms but also play a crucial role in predicting prognosis, planning management, and determining appropriate treatment strategies. For example, despite advances in the understanding of aneurysm pathophysiology and technological developments, aneurysms that are larger than 10 mm, have a wide neck, an unfavorable dome-to-neck ratio (<2), are located in the posterior circulation, and have a fusiform configuration continue to present significant therapeutic challenges, with over 20% of such cases not responding well to even the most advanced endovascular or surgical treatments available [43,44].

The current trend in IA size classification now favors the definitions proposed by the Japanese UCAS study: small (<5 mm), medium (5–10 mm), large (10–25 mm), and giant (>25 mm). This size classification system is recommended based on natural history data for IAs, as it more accurately encompasses higher-risk demographics. Additionally, it is suggested that a wide-neck aneurysm be defined as one with a neck diameter greater than 4 mm or a dome-to-neck ratio (DNR) less than 2, until further research provides evidence to support lowering the DNR threshold. The appropriateness of these definitions for IA size classification remains under evaluation and is a subject of ongoing discussion and research [45].

Aneurysms are classified based on their location within the brain’s vascular system. In the anterior circulation, aneurysms may be located at the anterior communicating artery (AComm), specifically at the junction of the anterior cerebral arteries, as well as at various segments of the internal carotid artery (ICA). Within the ICA, aneurysms may occur at the ophthalmic segment near the origin of the ophthalmic artery, at the posterior communicating artery (PComm), where it branches from the ICA, and at the bifurcation, where the ICA divides into the middle cerebral artery (MCA) and anterior cerebral artery (ACA). Middle cerebral artery (MCA) aneurysms typically arise at the bifurcation or trifurcation points of the MCA.

In the posterior circulation, aneurysms may be located on the basilar artery, either at its bifurcation or along its length, as well as on the vertebral arteries where they converge to form the basilar artery. Posterior inferior cerebellar artery (PICA) aneurysms are typically found at the junction of the vertebral artery and PICA. Additionally, peripherally located aneurysms involve branches of the cerebellar arteries, including the superior cerebellar artery (SCA), anterior inferior cerebellar artery (AICA), and posterior inferior cerebellar artery (PICA).

AComm aneurysms are the most prevalent type of IA, comprising 23–40% of all IA and 12–15% of unruptured cases. These aneurysms are particularly common among patients under 30 years of age. Due to their distinct anatomical and hemodynamic characteristics, AComm aneurysms have a higher likelihood of rupture compared to other types of IA [46,47,48]. The anatomical features of an AComm aneurysm significantly influence the choice between microsurgery and interventional embolization. Factors such as the aneurysm’s size and orientation play a crucial role in determining the complexity of the surgery and selecting the most appropriate surgical approach. Furthermore, the presence of calcification and intra-aneurysm thrombosis necessitates specific precautions during surgical clipping and may increase the risk of complications [49,50].

The anatomical structure of the bilateral A1 and A2 segments is critical in the formation of AComm aneurysms. The development of multiple aneurysms at the AComm is influenced by several factors, including hemodynamic stress. The diameters of these segments and their proportional relationships are closely associated with the formation and rupture risk of aneurysms. For instance, the diameter ratio between the A1 and A2 segments is significantly correlated with the likelihood of aneurysm rupture. During clipping surgeries or interventional embolization, the diameters and anatomical relationships of the bilateral A1 and A2 segments must be carefully considered to determine the optimal side for surgical clipping, assess the operation’s difficulty, and evaluate the potential need for stenting during embolization [51].

Previous studies have identified the presence of a dominant A1 segment and the dysplasia or deficiency of the contralateral A1 segment as key factors in the formation of AComm aneurysms. Additionally, research has shown that smaller A2 diameters and larger A1/A2 diameter ratios are associated with an increased likelihood of AComm aneurysm formation and rupture [51]. The angle between the A1 and A2 segments also plays a role in aneurysm formation and rupture risk. It was found that a smaller angle between these segments increased the probability of aneurysm development, particularly in patients with a dominant A1 segment [52].

These findings indicate that the absence or dysplasia of the A1 segment is closely associated with aneurysm formation. Research found that 49.8% of patients with AComm aneurysms exhibited hypoplasia of the A1 segment, a condition strongly correlated with anterior cerebral artery infarcts and adverse prognosis following aneurysm clipping. Multivariate analysis further identified A1 segment hypoplasia as an independent risk factor for poor prognosis after aneurysm clipping [53,54].

Anatomical variations, such as the infraoptic course of the A1 segment, are particularly noteworthy due to their significant impact on the occurrence and management of aneurysms [55]. In 2008 appeared the first classification of the relationship between the bilateral A2 segments in cases of superiorly projecting AComm aneurysms into two categories: the open A2 plane side (where the A2 segment near the aneurysm body is more posterior) and the closed A2 plane side (where the A2 segment is more anterior) [49]. Surgical procedures performed on the closed A2 plane side present challenges in exposing the aneurysm neck, often necessitating the removal of the gyrus rectus and manipulation of the A2 segment. These actions increase the risk of residual aneurysm necks and postoperative complications. Conversely, it was suggested that surgery performed on the open A2 plane side is both easier and safer for aneurysm neck exposure, thereby reducing the likelihood of postoperative complications [49].

## 4. Diagnostic Techniques and Imaging Tools

Magnetic resonance imaging (MRI) is highly effective for visualizing soft tissue structures; however, certain patients are contraindicated for this imaging modality. These contraindications include the presence of medical implants such as pacemakers, metal implants, or conditions like severe claustrophobia. In cases of claustrophobia, anesthesia may be administered to facilitate the procedure. Recent advancements, including 4D MRI and 3D contrast-enhanced MRI, have demonstrated promising potential in enhancing diagnostic accuracy and improving the follow-up of cerebral aneurysms and vascular abnormalities [56]. While computed tomography angiography (CTA) is generally preferred as the initial diagnostic test for ruptured aneurysms, magnetic resonance angiography (MRA) can be used to confirm the diagnosis in later stages. Although CTA is effective in the early stages, its utility diminishes in patients with diffuse subarachnoid hemorrhage, severe anemia, or minimal bleeding that is absorbed into the cerebrospinal fluid.

Furthermore, digital subtraction angiography (DSA) is regarded as the gold standard for detecting IA due to its superior accuracy, though it is not considered cost-effective as an initial diagnostic test [57]. As time progresses, identifying a ruptured aneurysm becomes increasingly challenging due to the diffusion of blood throughout the intracranial cavity. The location of blood collections within the intracranial cavity may help indicate the site of the original injury. For instance, ruptures of middle cerebral artery aneurysms often result in blood accumulating in proximal fissures such as the Sylvian fissure. Despite its invasive nature, DSA remains a valuable tool for radiologists, aiding in both diagnosis and guiding the appropriate treatment [58].

Combining CTA with digital subtraction angiography (DSA) provides the most comprehensive imaging for IA, allowing detailed visualization of flow patterns and aneurysm characteristics. Imaging is critical for the detection and characterization of aneurysms, as it can reveal essential details such as the aneurysm’s location, size, morphology, and geometry. These factors are crucial in determining the appropriate therapeutic strategy, whether that involves surgical intervention or conservative management. DSA, a fluoroscopic technique utilizing iodine contrast, produces high-resolution images of intracranial blood vessels by digitally subtracting surrounding tissues. It is considered the gold standard for imaging IA due to its superior spatial resolution, specificity, and sensitivity, enabling precise determination of morphological characteristics like size and neck diameter [59,60].

Newly developed 3D navigation systems have shown substantial benefits in aneurysm surgery. Neuronavigation enables the use of minimally invasive techniques, allowing surgeons to reach aneurysms more quickly while minimizing cortical damage. In a study using neuronavigation in 12 cases of distal anterior cerebral artery aneurysms, results showed safer surgeries with real-time imaging, smaller craniotomies, and no complications. Furthermore, neuronavigation has proven particularly advantageous in identifying distal middle cerebral artery aneurysms associated with intracerebral hematoma following rupture, enhancing surgical precision and outcomes [61].

The advent of 3D rotational angiography (3DRA) has further enhanced DSAs spatial resolution by eliminating imaging errors caused by the superposition of vascular structures, thereby enabling the visualization of small IAs (less than 3 mm). However, DSA is an invasive procedure and carries risks associated with the use of intra-arterial devices and iodine-containing contrast agents, including neurological complications (0.1–1%) and severe allergic reactions (0.05–0.1%) [62].

To address these concerns, several non-invasive imaging techniques, such as CTA, have been developed. CTA offers specificity and sensitivity levels that nearly match those of DSA for detecting IAs larger than 3 mm [sensitivity: 93.3–97.2%; specificity: 87.8–100%] [63]. However, CTA is less effective at detecting small IAs located near the skull bone due to the similar absorption of ionizing rays by calcium and iodinated contrast agents [sensitivity: 61%] [64]. Techniques like match mask bone elimination (MMBE) have been developed to enhance specificity by eliminating bone-induced signals, but these require longer exposure to ionizing radiation and are sensitive to patient movement. Dual-energy CTA (DE-CTA) has improved material differentiation capabilities, reducing artifacts from bony structures without the drawbacks associated with MMBE [65,66].

Magnetic resonance angiography (MRA) offers a less invasive alternative to traditional imaging techniques, as it does not utilize X-rays. MRA sequences, such as time-of-flight MRA (TOF-MRA) and non-enhanced magnetization-prepared rapid acquisition gradient echo (MPRAGE), have garnered significant interest due to their ability to visualize IA without the use of contrast agents, thus avoiding the health risks associated with iodinated agents. TOF-MRA at 1.5 and 3 Tesla (T) is commonly employed for visualizing IAs, with greater sensitivity and accuracy achieved at 3T [sensitivity: 1.5T = 53.6%, 3T = 76.6%; accuracy: 1.5T = 84%, 3T = 91.9%]. However, artifacts may occur in cases of turbulent or low blood flow, which are common in large or coiled aneurysms. Gadolinium-enhanced MRA (GE-MRA) can be utilized as a flow-independent method that also avoids the need for contrast agents [67,68,69,70].

Both TOF-MRA and GE-MRA demonstrate a sensitivity of 95% when compared to DSA [14]. Recently, the use of 7T MRA has been evaluated for studying IAs. Although 7T MRI is not yet widely available, it shows significant potential for detecting IAs and providing detailed anatomical descriptions, making it a valuable tool for IA follow-up. The combination of 7T 3D-TOF and MPRAGE has been shown to delineate unruptured IAs as effectively as DSA. Additionally, intracranial black blood vessel imaging (MR-IBBVI), a novel MRA sequence based on blood signal suppression, offers higher sensitivity and specificity than TOF-MRA, regardless of aneurysm size [sensitivity: MR-IBBVI = 94.5%, TOF-MRA = 62.7%; specificity: MR-IBBVI = 94.5%, TOF-MRA = 92%, both compared with DSA] [71,72,73].

However, classical imaging techniques, including those mentioned above, do not allow for the observation of vessel wall remodeling, which is a critical feature of IAs that are progressing toward rupture. Currently, no imaging technique is capable of visualizing the disruption of the elastic lamina or the thinning of the media. Optical coherence tomography (OCT), a widely utilized technology in the field of ophthalmology, is currently being refined for uses within the cranium. OCT functions by exploiting the variable reflective characteristics of tissues to near-infrared light. A catheter is inserted into the specific blood vessel, and high-resolution 2D cross-sectional pictures are obtained (with a resolution ranging from 1 to 15 μm). Research has demonstrated that Optical Coherence Tomography (OCT) is capable of detecting disruptions in the layers of IA, where the borders between the intima and media layers become blurred, as opposed to a normal vessel wall. Furthermore, Optical Coherence Tomography (OCT) can be utilized in real-time to observe and verify the accurate placement of intrasaccular devices during endovascular procedures [74,75]. The development of these imaging techniques has the potential to greatly improve the current methods for assessing the risk of IA rupture. Currently, the risk assessment is mostly based on the shape of the IA, as determined by the available imaging technologies.

The significance of hemodynamic stresses in growth, enlargement, and rupture of IA has been well-established. Important hemodynamic parameters consist of wall shear stress (WSS), which reflects the tangential frictional force exerted by blood flow on the vessel wall; oscillatory shear index (OSI), which quantifies the direction and magnitude of flow variations throughout a cardiac cycle; relative residence time (RRT), which suggests the movement of blood flow in time at the aneurysm wall; and flow patterns. Presently, there is widespread recognition that high wall shear stress (WSS) is a contributing factor in the formation of IA. Nevertheless, its impact on aneurysm rupture is not as well understood, as equally high and low WSS can result in detrimental remodeling of the aneurysm wall. The presence of high wall shear stress (WSS) is thought to initiate a pathogenic response mediated by mural cells, whereas low WSS is linked to an inflammatory response mediated by immune cells. Nevertheless, the occurrence of IA rupture is more commonly linked to elevated OSI, extended RRT, and intricate flow patterns, which cannot be easily observed using the clinical imaging techniques discussed in the morphological imaging part of this study [76,77,78].

Computational fluid dynamics (CFD) is a commonly employed method for investigating hemodynamic factors, and it heavily depends on detailed 3D datasets with high resolution. Computational Fluid Dynamics (CFD) employs the inherent properties of IA, such as their dimensions, position, aspect ratio, and size ratio, to determine Wall Shear Stress (WSS), Oscillatory Shear Index (OSI), flow velocity, and Residence Time Ratio (RRT). CFD results are significantly affected by the selection of imaging modality. However, there is no commonly accepted imaging modality that is considered the most accurate for CFD calculations. Although CFD is effective in calculating hemodynamic parameters and improving our understanding of IAs, it has several limitations. These include the assumption that blood behaves as a Newtonian fluid and arteries are rigid structures, as well as the lack of a standardized protocol. These limitations have been reviewed in previous studies [76,77,78,79,80,81].

In clinical practice, 3D rotational angiography (3DRA) is considered the gold standard for detecting and defining the static characteristics of aneurysms. However, there is no consensus on the best method for assessing dynamic features. Typically, a combination of 2D and 3DRA is employed to evaluate cerebrovascular blood flow. 2D digital subtraction angiography (2D-DSA) provides information on flow dynamics during the passage of a contrast agent, while 3DRA offers static anatomical details. This combination has led to the development of 4D-DSA, or time-resolved 3DRA, which integrates 2D-DSA and 3DRA. This method utilizes 3D images obtained from conventional 3DRA while retaining temporal information, thereby allowing for the visualization of contrast agent influx and efflux from any angle [82,83].

An analysis reviewed several studies that successfully detected and quantified IA wall deformation across different frames with high spatial and temporal resolutions (ranging from 35 to 165 ms and 0.2 mm, respectively). Although most applications of 4D digital subtraction angiography (4D-DSA) have been explored in the context of arteriovenous malformations, only one study has qualitatively assessed its effectiveness in detecting IA flow patterns, reporting excellent visualization in 27.7% of IAs and fair visualization in 72.3%. Additionally, it was demonstrated that 4D-DSA is as reliable as 3D rotational angiography (3DRA) for computational fluid dynamics (CFD) analysis, showing no significant differences in flow velocity or wall shear stress (WSS) calculations [84]. With its high spatial resolution (voxel volume = 0.008 mm^3^), 4D-DSA provides anatomical characterization of IAs comparable to that of the gold standard 3DRA. Therefore, while 4D-DSA still requires refinement for the direct quantification of blood hemodynamics, its spatial resolution supports robust CFD analysis alongside morphological characterization of IAs [83,84,85].

Traditionally, blood flow visualization with magnetic resonance imaging (MRI) has utilized the phase-contrast method to assess unidirectional flow in a 2D space. This method has since evolved into 3D time-resolved phase-contrast MRI, also known as 4D-MRI. This advanced imaging technique quantifies blood flow velocity directly in 3D, enabling the modeling of flow patterns and the quantification of hemodynamic parameters such as WSS, oscillatory shear index (OSI), and vorticity. In 2020, two comprehensive reviews were published on the capabilities of 4D-MRI in studying IA hemodynamics. These reviews indicate that 4D-MRI, often compared to CFD, reliably depicts intra-aneurysmal flow patterns across various IA morphologies. Nevertheless, 4D-MRI is subject to limitations, namely in relation to its spatial and temporal resolution, which can have an effect on the precision of hemodynamic parameter calculations [86,87].

For example, voxel sizes in 4D-MRI range from 0.43 × 0.43 × 0.43 mm^3^ to 1 × 1 × 1.6 mm^3^, whereas computational fluid dynamics (CFD) simulations typically use voxel sizes around 0.1 mm. As a result, wall shear stress (WSS) values derived from 4D-MRI tend to be of lower magnitude, although the localization of WSS remains comparable. Another limitation of 4D-MRI in clinical settings is its lengthy acquisition time, which can range from 5 to 30 min depending on the magnetic field strength and acquisition protocol. To mitigate this, an accelerated high spatiotemporal resolution 4D-7T-MRI technique has been proposed, which provides accurate quantitative flow measurements within a reduced acquisition time of 10 min [56,88,89].

Compared to classical CTA, which may require longer acquisition times or multiple acquisitions over a period, 4D-CTA captures the influx and efflux of contrast agents and morphological changes in IA within a cardiac cycle when acquisition is ECG-gated. 4D-CTA is primarily utilized in evaluating hemorrhagic or ischemic stroke and vascular malformations, and it has been suggested as a potential replacement for the gold standard 3D digital subtraction angiography (DSA) in follow-up imaging. This is because 4D-CTA produces accurate IA geometries and reliable CFD results comparable to those obtained with 3D rotational angiography (3DRA) [90].

Beyond these conventional hemodynamic parameters, aneurysmal pulsatility has emerged as an important dynamic parameter for assessing IAs. Increased wall motion is believed to be associated with reduced aneurysm wall stability and an increased risk of rupture. This pulsation, which includes both the global pulsation of the aneurysm and the movement of focal parts such as blebs, must be distinguished from the physiological cerebrovascular movement that occurs during the cardiac cycle. Due to the rapid and low magnitude nature of these pulsations, developing an accurate imaging modality poses significant challenges. A study using 7 T MRI to quantify volume pulsation demonstrated insufficient accuracy due to multiple imaging artifacts. The most common technique for studying aneurysmal pulsation is 4D-CTA, which achieves spatial resolution comparable to the movement of the aneurysm under study (with high-resolution CT scans providing 0.25 mm resolution and standard scans offering 0.6–0.8 mm resolution). This technique has been reported to effectively measure aneurysm pulsation in IAs larger than 5 mm in vivo [91,92].

Another risk factor that warrants consideration is the Coanda Effect, which may offer insight into the behavior of blood flow in specific scenarios. The understanding of brain aneurysms has been advanced through the study of convex structures within blood vessels. Convex structures, such as cholesterol plaques, can develop inside arteries, influencing blood flow dynamics. The Coanda Effect describes the tendency of a fluid flow to adhere to a nearby surface. In the context of blood flow within arteries, this effect causes the blood to adhere to the wall opposite the convex structure, such as a plaque. This interaction reduces the resistance of the affected arterial wall, eventually leading to the formation of a concave. As the Coanda Effect continues to direct blood flow towards the concavity, a cycle ensues that can ultimately result in the formation of a saccular aneurysm [93] (Table 1).

## 5. Aneurysm Management Options and Decision-Making

Selecting an appropriate treatment strategy for IA is a complex and nuanced decision that requires careful consideration of various factors. Therapeutic decision-making must balance the potential risks and benefits of conservative management versus aggressive intervention, with decisions tailored to the individual patient’s circumstances and the resources available at the treating hospital. The primary options for aneurysm management include open microneurosurgery and endovascular techniques, each offering distinct advantages and drawbacks.

Several key factors influence the decision-making process for aneurysm treatment. These factors include the rupture status of the aneurysm, whether it is symptomatic or asymptomatic, and the presence of a family history or genetic predisposition to aneurysms. Additional risk factors such as hypertension, smoking, alcohol use, and drug use are also critical considerations. The size and location of the aneurysm—whether it is situated in the anterior or posterior circulation, within the anterior or posterior communicating segment, or in extradural versus intradural locations—are significant determinants in the treatment approach. High-risk aneurysm characteristics, such as changes in size or morphology over time, the emergence of new neurologic or cranial nerve symptoms, irregularities, the presence of daughter lobes, and a history of prior subarachnoid hemorrhage, also play a pivotal role in the decision-making process.

Moreover, the patient’s age, functional status, comorbidities, life expectancy, vascular anatomy, and the anticipated risk of surgical or endovascular treatment are essential considerations when determining the most appropriate course of action. The complexity of these factors underscores the importance of a personalized approach in the management of IA, with treatment strategies carefully tailored to optimize patient outcomes.

### 5.1. Neurosurgical Techniques

The primary microsurgical techniques for managing IA focus on placing a clip across the neck of the aneurysm to exclude it from circulation while preserving the patency of the parent vessel. Alternative surgical options include proximal arterial occlusion, bypass techniques, aneurysm wrapping, or trapping, which involves combined proximal and distal vessel occlusion.

Attaining adequate neck exposure is crucial in aneurysm clipping procedures. Over time, the advancement of different surgical techniques for aneurysms located at the base of the skull has greatly decreased the distance between the surgeon and the aneurysm. This has resulted in less exposure and pulling of the neurovascular tissue, while also improving the surgeon’s ability to perform the procedure. After the dura is opened, the main goal is to achieve control and release of the aneurysm neck with minimal dissection. Performing dissection in close proximity to the aneurysm poses the danger of rupture, so the most secure method is to maneuver around the parent artery. It is essential to identify the parent artery and conduct a meticulous dissection towards the aneurysm neck along this blood supply.

The approach to obtaining proximal control is contingent upon the specific location of the aneurysm. Dissecting the complete parent artery from its origin is typically unnecessary. In the case of an AComm aneurysm, the A1 segment near the optic nerve is usually identified to establish proximal control. When dealing with a middle cerebral artery aneurysm, especially with the smaller Sylvian approach, achieving proximal control provides only a certain level of safety. However, to ensure complete control, it is necessary to manage all the arteries that supply blood to and drain blood from the aneurysm.

Once the parent artery is accessed, the approach to the aneurysm neck should be made along this artery. Both sides of the aneurysm neck must be separated from the arterial branches sufficiently to allow the clip blades to pass smoothly, typically requiring a clearance of at least 3 mm. At this stage, it is important to avoid contact with the aneurysm dome. Complete separation of the aneurysm neck from the arteries is necessary to allow the dissector to pass through. Temporary clipping is often a useful technique during the clipping of the aneurysm.

After adequate dissection, the next step is to select the clip that best fits the aneurysm neck, using the angiogram as a guide. The clip length should ideally match the neck diameter multiplied by π/2. If this measurement appears too small during the procedure, it may indicate that the perceived neck includes a nearby brain artery. Any bleeding from the aneurysm should be controlled immediately. For smaller aneurysms, this can often be managed by applying a cotton pad with gentle compression. In cases of larger leaks or aneurysms, temporary clipping of all arteries supplying the aneurysm may be necessary [94].

It is crucial to ensure that the clip fully spans the aneurysm neck without entrapping any perforating arteries. Small dogears at the base of the aneurysm should be addressed with additional small clips to ensure complete occlusion.

Preoperative imaging plays a vital role in selecting the appropriate surgical approach. Factors such as the aneurysm’s size, projection, and its relationship to nearby structures must be carefully evaluated to optimize visualization and minimize surgical risks. A thorough understanding of the aneurysm’s position relative to surrounding anatomical features is essential for successful surgery [95].

### 5.2. Endovascular Techniques

Endovascular therapy, particularly the coiling of cerebral aneurysms, has become the primary treatment modality for IA in many neurovascular centers. This shift is largely due to the progressive refinement of coiling devices and techniques, as well as evidence demonstrating significant benefits for patients with a good preoperative clinical grade.

The primary goal of coiling is to achieve dense packing within the aneurysm sac to induce rapid coagulation, effectively isolating the aneurysm from active circulation. The geometry of the aneurysm is a critical factor in determining the appropriate treatment approach and its likely outcome. Unassisted coiling is typically well-suited for IA that exhibit favorable anatomical characteristics, such as an optimal neck width, a favorable dome-to-neck ratio, an appropriate aspect ratio, and a favorable relationship with branch vessels. The conventional techniques include balloon-assisted coiling (BAC) and stent-assisted coiling (SAC). However, more complex aneurysms with unfavorable anatomy may require additional techniques to achieve successful treatment. These adjunct techniques provide the necessary support to manage challenging geometries and ensure effective isolation of the aneurysm [96].

Flow-diverting (FD) stents are another advanced option, designed to divert blood flow away from the aneurysm, promoting stasis and natural thrombosis within the aneurysm sac while also providing a scaffold for endothelial growth.

Endovascular coiling continues to be a viable and effective treatment option for both ruptured and unruptured aneurysms, often serving as an alternative to surgical clipping, which is associated with higher morbidity and mortality rates. Advances in coil embolization techniques now include balloon-assisted methods and the use of intracranial stents to enhance coil packing, maintain parent artery patency, and reduce the risk of coil herniation [97]. Additionally, significant improvements in coil properties have been made to increase aneurysm occlusion rates, further enhancing the efficacy of this treatment approach [98].

Wide-neck cerebral aneurysms pose significant technical challenges for endovascular coiling. These aneurysms are generally defined by a dome-to-neck ratio of less than 2 or a neck diameter of 4 mm or greater. The primary challenges in coiling wide-neck aneurysms include the propensity for coils to herniate into the parent vessel, difficulty in clearly defining the interface between the aneurysm neck and the parent vessel, and the need to protect vessel branches located at the aneurysm neck [99]. In order to address these difficulties, additional devices such as intracranial stents and balloons have been created. Balloon-assisted coil embolization decreases the likelihood of coil displacement into the main artery and provides immediate control near the source in case of an aneurysm rupture during the procedure. Stent-assisted coiling enhances the density of coil packing, which is crucial for treating aneurysms that are wide-necked, massive, or enormous in size. This method greatly enhances the rates of obliteration and decreases the probability of aneurysm regrowth [97,100,101].

Balloon remodeling during endovascular coiling involves the temporary inflation of a balloon catheter across the aneurysm neck during coil placement. This technique enhances coil packing density, and the balloon is deflated and removed at the conclusion of the procedure. In the event of an aneurysm rupture during coiling, the balloon can temporarily occlude the parent artery, providing crucial control. Originally, balloon remodeling was performed using a single low-compliance balloon, which was mainly suited for sidewall aneurysms. However, more compliant balloons are now available, including Hyperform, HyperGlide (single lumen), TransForm (single lumen), and Scepter (dual lumen) [97,102]. Despite initial concerns regarding blood stasis and thrombus formation during balloon inflation, studies have shown that the safety profile of balloon-assisted coiling is comparable to that of conventional coiling [103,104].

The TransForm occlusion balloon catheter (Stryker Neurovascular, Fremont, CA, USA) represents a significant advancement in balloon microcatheter design. This catheter features a single lumen that is compatible with 0.014-inch microguidewires and is available in both compliant and super-compliant versions. Its micromachined hypotube design facilitates rapid inflation and deflation, enhances visibility, and reduces procedural times. An early clinical study involving 23 aneurysms treated with the TransForm catheter reported a complete aneurysm occlusion rate (Raymond Roy Classification I) of 78%, with no serious complications observed [105]. A systematic review researched its use in 63 bifurcation aneurysms across Europe and the USA, concluding that it is “safe and probably effective” for treating intracranial bifurcation aneurysms, although larger sample sizes are necessary to draw definitive conclusions [106]. More recently, it was reported early post-market results for the treatment of 54 aneurysms across 13 US centers, further contributing to the growing body of evidence supporting the efficacy and safety of the TransForm catheter [107].

### 5.3. Relevant Case Studies

The primary objective of IA treatment is to disconnect blood flow from the parent artery to the aneurysm lumen, ultimately achieving complete aneurysm occlusion. Over the past century, and particularly in the last 30 years, treatment approaches have evolved significantly, providing neurosurgeons with a range of strategies to choose from.

Europe and South America were the first regions to have clinical trials and use flow diverters (FDs) for treating aneurysms. Flow diverter devices, initially released in Europe in 2007 and later in the United States four years later, provide an efficient endovascular therapy for aneurysms that are big (>10 mm) and have a wide neck (>4 mm), particularly those found in the siphon region of the internal carotid artery (ICA) [108]. These devices are specifically advised for aneurysms situated in the internal carotid artery (ICA), extending from the petrous to the superior hypophyseal segments. Flow diverters are metallic implants with low porosity that are similar to stents. They are implanted in the parent artery of the aneurysm. All patients who receive flow diverters are required to undergo dual antiplatelet therapy.

Flow diverters are being used more frequently, especially for patients who have a high risk during surgery. Endovascular techniques have emerged as the favored method of treating many aneurysms in the posterior circulation, which pose a greater risk of rupture and compressive symptoms compared to aneurysms in the anterior circulation. Furthermore, flow diversion is used as an alternate treatment option when conventional surgical methods are not recommended. It also allows for the sequential treatment of several aneurysms, resulting in positive outcomes and low rates of complications and death [109].

Flow diverters function by reducing blood flow within the aneurysm and redirecting it to the parent vessel. The Silk device (Balt) and the Pipeline Embolization Device (PED; ev3/Covidien) received CE (Conformité Européenne) approval in 2008. The early clinical use of the Silk device revealed potential hemorrhagic and thromboembolic complications, particularly in electively treated asymptomatic patients. Initial unfamiliarity with the device and the inability to predict these complications led to some early disappointment with FD treatment [110,111]. However, the perception of flow diverters improved significantly following the publication of the results from the Pipeline Embolization Device for the Intracranial Treatment of Aneurysms (PITA) trial. This multicenter, single-arm prospective study documented the experience of neurointerventionalists using PEDs to treat 31 predominantly large, wide-necked ICA aneurysms across three European centers and Argentina, achieving a complete occlusion rate of 93% at six months with a low complication rate of 6.5% [112].

The first-generation Pipeline Embolization Device (PED) gained FDA approval in 2011 following the results of the Pipeline for Uncoilable or Failed Aneurysms (PUFS) trial. This prospective, multicenter, single-arm trial, which commenced in 2008 and concluded in 2014, involved 109 large and giant (≥10 mm) wide-necked internal carotid artery (ICA) aneurysms. The trial reported impressive complete occlusion rates of 86.8% at one year, 93.4% at three years, and 95.2% at five years, with low rates of thromboembolic complications (5.6%) and retreatment (5.7%) [113,114,115]. The second generation of the PED, known as Pipeline Flex, received FDA approval in 2015. This updated device retained the original PED design but introduced an enhanced delivery mechanism featuring a resheathing capability, which improved maneuverability and reduced both procedure and fluoroscopy times [116].

A larger single-center study involving 491 anterior circulation aneurysms treated with the PED demonstrated a 78% complete occlusion rate at 12 months. The study identified several predictors of nonocclusion, including larger aneurysm size, incorporation of a branch vessel into the aneurysm dome or neck, and male sex [117]. Similarly, a multicenter study, which examined 523 PED-treated aneurysms, reported a 76.6% complete occlusion rate at 12 months. In this study, older age (>70 years), aneurysm size ≥ 15 mm, and fusiform morphology were identified as independent predictors of nonocclusion [118]. Fusiform morphology was also recognized as a predictor of failure to occlude in another study [119].

Flow diverter (FD) treatment, while generally effective, is not without risks, particularly within the first 30 days post-procedure. Complications may include thromboembolic or ischemic events, intracranial hemorrhage (due to parent artery injury or aneurysm rupture), and FD stent malposition or migration. Hyperacute or acute in-stent thrombosis, distal vessel occlusions, and spontaneous embolization (SBO) are more common in patients with complex aneurysms or tortuous parent vessel anatomies. Treatment options for these complications include glycoprotein IIB/IIIa inhibitors, intra-arterial fibrinolytics, or mechanical thrombectomy, though these interventions carry a risk of intracranial hemorrhage. Additional procedural complications, such as parent vessel perforation, may also occur and require interventions such as heparin reversal, blood pressure control, or, in extreme cases, parent vessel sacrifice [120,121] (Table 2).

### 5.4. Hybrid Microsurgical and Endovascular Approach in the Management of Multiple Cerebral Aneurysms

While the majority of IA can be effectively treated using either microsurgical or endovascular techniques alone, a subset of patients with complex or multiple aneurysms may benefit from a combined approach. The primary objective of any IA treatment is to achieve complete occlusion of the aneurysm while preserving the patency of the parent arterial flow [122]. To meet this objective, a combined approach that incorporates both microsurgical and endovascular techniques may be advantageous, as it can minimize risk while maximizing treatment efficacy and outcomes. For example, one-stage endovascular coiling and microsurgical clipping of multiple remote aneurysms on opposite sides can be particularly effective when a middle cerebral artery (MCA) aneurysm, suitable for clipping, coexists with a difficult-to-reach contralateral aneurysm that is more amenable to coiling. In this context, microsurgical and endovascular techniques should be viewed as complementary rather than competing, and a combined management strategy can positively influence patient outcomes [123,124].

Some experts advocate for one-stage or hybrid surgeries in the treatment of multiple IA, noting that the surgical risk for such procedures is only slightly higher than that for single aneurysms [125,126]. For instance, the clipping of multiple ipsilateral MCA aneurysms in a single surgery remains a preferred procedure due to its low morbidity, mortality, and recurrence rates. However, the expansion of endovascular techniques provides robust alternatives for these aneurysms, especially in cases where surgical clipping is less suitable. Endovascular methods have proven to be a well-established alternative in such scenarios [127].

One significant advantage of the endovascular approach is its superior ability to accurately determine the rupture site, thereby reducing the risk of misdiagnosis or incorrect localization. Moreover, endovascular procedures allow for the simultaneous occlusion of all aneurysms, regardless of their rupture status, within a single session, ensuring that the bleeding aneurysm is not left untreated [128]. Although ischemic and hemorrhagic complications are more frequent in endovascular treatment compared to clipping, this approach is particularly suitable for high-risk aneurysms. As a result, endovascular treatment is recommended for contralateral aneurysms and those that are technically challenging to clip.

## 6. Advances in Technology and Technique

Liquid embolization, initially used primarily for treating arteriovenous malformations and fistulae, has also been employed in the management of IA. Around the time when stand-alone coiling procedures first emerged, some clinicians began incorporating liquid embolization materials alongside other space-occupying embolic agents. This approach was informed by an evolving understanding of the mechanisms underlying aneurysm exclusion from intracranial circulation, moving beyond the previously supported electrothrombosis model. In the 1990s, a study showed 19 giant aneurysms treated using a combination of detachable balloons, occlusion coils, and ethylene vinyl alcohol copolymer liquid. However, concerns about the generation of emboli and the use of dimethyl sulfoxide (DMSO) in the cerebral vasculature led to the temporary abandonment of these techniques [129,130].

The development of newer materials, such as the more viscous Onyx HD-500—composed of an ethylene vinyl alcohol copolymer dissolved in a DMSO solvent—marked a significant advancement. The Cerebral Aneurysm Multicenter European Onyx (CAMEO) trial, published in 2004, demonstrated that Onyx HD-500 embolization achieved superior occlusion rates compared to coil embolization in previously treated aneurysms of similar types [131].

Despite these favorable results in the hands of skilled operators, the complexity and time-consuming nature of the procedure contributed to its decline. The procedure often involved repeated balloon inflations and deflations, necessitating delicate “seal tests” with contrast injection. These steps could significantly prolong the procedure, especially if contrast injection disrupted the embolic material. Balloon inflation had to be carefully managed to minimize the risk of cerebral ischemia, while inadequate inflation posed the risk of emboli formation and improper precipitation of the liquid embolic material. Additionally, balloon migration during the serial deflation/reinflation cycles often required retrieval and redeployment, further extending the duration of the procedure.

In the modern era of flow diversion and other advanced treatments, liquid embolization remains a viable strategy for specific cases, particularly in patients with incomplete aneurysm occlusion, persistent high-risk features, or nickel allergies. However, the impact of nickel allergies on related complications has been a subject of ongoing debate [132].

Although the transition from stand-alone coiling to stent-assisted coiling has become evident for the treatment of many aneurysms, no initial studies explicitly demonstrated the inferiority of intrasaccular liquid embolization compared to newer treatment strategies. However, long-term follow-up revealed that the durability of intrasaccular liquid embolization was suboptimal [133]. In the current era of flow diversion and intrasaccular flow disruption, the use of intrasaccular liquid embolization has become limited and is no longer commercially available. This is despite its theoretical advantages, such as the potential to fill 100% of an aneurysm, provide a surface for endothelialization, and effectively occlude giant aneurysms [134].

The limited applicability of flow diverters (FDs) in acutely ruptured or bifurcation aneurysms presents ongoing challenges in the endovascular treatment of these complex cases. The conventional methods, including balloon-assisted coiling (BAC) and stent-assisted coiling (SAC), though technically demanding, are associated with higher rates of incomplete occlusion, recanalization, retreatment, intraoperative and postoperative complications, and bleeding due to the necessity of antiplatelet therapy. These challenges underscore the need for enhanced operator skills and have driven increased research focus on the treatment of complex bifurcation aneurysms [135].

The pCONus device, which has been extensively utilized, consists of a self-expanding, laser-cut stent with a distal crown of four petals that are deployed within the aneurysm and a base with six polyamide fibers at the neck. This design facilitates stable coil placement by providing a mechanical barrier at the aneurysm neck. The second-generation pCONus device, which features six distal petals and omits the polyamide fibers, is better suited to accommodate steep angles between the parent vessel and the aneurysm sac, offering greater metal coverage inside the sac to aid in coiling [136].

Similarly, the eCLIPs device, a laser-cut, non-circumferential device, includes an “anchor” for the neck and a “leaf segment” with movable ribs that can be delivered through a coiling microcatheter. The leaf segment offers 23–42% metal coverage over the aneurysm neck, functioning as a flow disruptor and a scaffold for endothelial growth. The second-generation eCLIPs device is self-expanding, microcatheter-deliverable, fully retrievable, and self-orienting, offering a refined approach to managing complex aneurysms [137].

## 7. Future Directions

Selecting the appropriate treatment for IA is a multifaceted decision that depends on numerous factors, including patient characteristics, the specifics of the aneurysm, available hospital resources, and the expertise of the surgeon. Age is particularly significant in determining the optimal management strategy. Prior to 1990, surgical clipping was the primary treatment for ruptured aneurysms. However, the introduction of Guglielmi Detachable Coils (GDC) provided a viable alternative. The International Subarachnoid Aneurysm Trial (ISAT) compared endovascular coiling with surgical clipping and found that coiling resulted in better disability-free survival at one year. Long-term follow-up data from ISAT indicated lower mortality rates with coiling, although there was a slightly higher risk of rebleeding [138].

Despite these findings, the ISAT results are somewhat limited by selection bias, as the study excluded many aneurysm types and primarily focused on patients in good clinical condition, making it difficult to generalize the outcomes. The Barrow Ruptured Aneurysm Trial (BRAT) sought to provide a more accurate reflection of real-world conditions. This trial found that coiling was associated with fewer poor outcomes one year post-treatment, although this difference was not statistically significant after three years [139].

For unruptured aneurysms, research also tends to favor coiling over clipping. For example, a study analyzed data from nearly 5000 patients and found that those who underwent surgical clipping faced higher risks of complications and less favorable outcomes compared to those treated with coiling. A large meta-analysis supported these findings, demonstrating higher rates of independence and lower mortality with coiling [140].

## 8. Conclusions

The evolution of endovascular aneurysm treatments has been a gradual process, with each new device addressing previous challenges and improving safety and efficacy. Innovations in this field have made these treatments more accessible and effective. For example, many clinicians now favor the transradial approach over the traditional transfemoral method due to its lower complications rates. Advances in microcatheters and microwires have enabled access to previously unreachable blood vessels, expanding the scope of endovascular interventions. Enhanced antiplatelet therapies have also reduced complications associated with certain procedures, while optimized follow-up protocols have minimized unnecessary visits, thereby lowering patient risks and improving overall care.

Looking ahead, future devices may leverage our growing understanding of aneurysm healing by incorporating active protein coatings designed to promote better healing within aneurysms. Endovascular coiling techniques continue to advance, with the development of new, low-profile stents and temporary bridging devices aimed at increasing the efficacy of coiling procedures. Innovations such as the Woven EndoBridge (WEB) device are showing great promise, and the use of 3D-printed vascular models is becoming increasingly popular for pre-procedural planning. These advancements are continually raising the standards for safety and effectiveness in aneurysm treatment.

Moreover, it is important to be noted that the cutting-edge technology should be integrated with vast anatomical knowledge in order to obtain great treatment results. For example, liquid embolization, initially used for arteriovenous malformations, has also been applied to IA. Integrating neuroanatomical knowledge with cutting-edge techniques has been crucial to the evolution of this procedure. Early approaches combined embolization with coiling, but neuroanatomical complexities, such as vessel architecture and blood flow patterns, led to complications like emboli formation and challenges with dimethyl sulfoxide (DMSO) use. The introduction of Onyx HD-500 improved occlusion rates, but the procedure’s complexity underscored the importance of anatomical precision. Today, liquid embolization is reserved for specific high-risk cases, but newer devices like pCONus and eCLIPs offer enhanced control, thanks to their ability to navigate the neurovascular anatomy more precisely. These innovations, informed by a deep understanding of cerebral vasculature, improve the effectiveness of treating complex aneurysms, demonstrating the critical role of neuroanatomy in advancing surgical practices.

## Figures and Tables

**Table 1:** Summary of the Diagnostic Techniques and Imaging Tools.

Imaging Technique	Description	Advantages	Limitations
Magnetic Resonance Imaging (MRI)	Effective for visualizing soft tissues. New techniques include 4D MRI and 3D contrast-enhanced MRI.	High resolution, good for soft tissues, non-invasive (in some forms).	Contraindicated in patients with implants, severe claustrophobia.
Computed Tomography Angiography (CTA)	First-line test for ruptured aneurysms uses X-rays and contrast agents.	High sensitivity and specificity for aneurysms > 3 mm.	Less effective for small aneurysms and near bones, requires ionizing radiation.
Digital Subtraction Angiography (DSA)	Gold standard for detecting intracranial aneurysms uses iodine contrast.	Superior accuracy and spatial resolution.	Invasive, high cost, potential for neurological complications.
3D Rotational Angiography (3DRA)	Advanced form of DSA with enhanced spatial resolution.	Visualizes small aneurysms (<3 mm) and eliminates imaging errors.	Invasive, carries risks with contrast agents.
4D DSA	Combines 3DRA with dynamic flow information.	High spatial resolution, useful for CFD analysis.	Not yet fully refined for quantifying blood hemodynamics.
Magnetic Resonance Angiography (MRA)	Non-invasive MRI technique without X-rays. Time-of-flight MRA (TOF-MRA) and gadolinium-enhanced MRA are common.	Avoids contrast agents, useful for patients contraindicated for CTA.	Artifacts may occur with turbulent or low blood flow.
7T MRA	Higher resolution MRI for detailed anatomical descriptions.	Effective for IA follow-up and morphology analysis.	Limited availability.
Optical Coherence Tomography (OCT)	Provides high-resolution 2D cross-sectional images of blood vessels.	Detects disruptions in aneurysm layers; real-time observation during procedures.	Invasive, limited use in neurology so far.
4D-MRI	Advanced MRI that visualizes blood flow velocity and models hemodynamic parameters.	Good for studying IA hemodynamics and flow patterns.	Lower resolution compared to CFD, long acquisition times.
Computational Fluid Dynamics (CFD)	Computational analysis of hemodynamic factors like WSS, OSI, and RRT.	Enhanced understanding of aneurysm dynamics.	Limited by assumptions (e.g., rigid arteries), lacks standardization.
4D-CTA	Uses CT to visualize contrast agent movement and morphological changes during the cardiac cycle.	Good for assessing aneurysms and strokes, potential CTA replacement.	Requires multiple acquisitions and longer exposure to radiation.

**Table 2:** Summary of the Relevant Case Studies.

Case Study	Description	Key Outcomes	Complications
Flow Diverters (FD)—Initial Use	First used in Europe (2007) for treating large and wide-neck aneurysms, especially in the siphon region of the internal carotid artery (ICA) [108].	Positive results in reducing blood flow within aneurysms, good outcomes in high-risk surgical patients [109].	Initial hemorrhagic and thromboembolic complications and unfamiliarity led to early disappointment [110,111].
Pipeline Embolization Device for Intracranial Treatment of Aneurysms (PITA) Trial	Prospective study treating 31 large ICA aneurysms with the Pipeline Embolization Device (PED) [112].	93% complete occlusion rate at 6 months, 6.5% complication rate [112].	Minimal complications and early success helped improve perception of FDs [112].
Pipeline for Uncoilable or Failed Aneurysms (PUFS) Trial	Large trial (109 patients) treating wide-neck ICA aneurysms with PED [113,114,115].	86.8% occlusion at 1 year, 95.2% at 5 years, with low complication (5.6%) and retreatment (5.7%) rates [113,114,115].	Thromboembolic events and low rates of retreatment were reported [113,114,115].
Pipeline Flex Device	Second-generation PED with improved delivery and resheathing capabilities [116].	Easier maneuverability, reduced procedure, and fluoroscopy times [116].	Same overall effectiveness as PED with enhanced usability [116].
Large Single-Center Study	491 anterior circulation aneurysms treated with PED [117].	78% complete occlusion rate at 12 months [117].	Nonocclusion predictors: larger aneurysm size, branch vessel involvement, male sex [117].
Multicenter Study on PED	Study on 523 aneurysms treated with PED [118].	76.6% complete occlusion rate at 12 months [118].	Older age, aneurysm size ≥ 15 mm, and fusiform morphology identified as nonocclusion predictors [118,119].
Complications of FD Treatment	Common risks within 30 days post-procedure [120,121].	Low rates of thromboembolic complications, managed with various interventions [120].	Complications include thromboembolic events, stent malposition, hyperacute thrombosis, and parent vessel perforation [120,121].

## Abbreviations

CFD	Computational flow dynamics
IA	Intracranial aneurysms
WSS	Wall shear stress
OSI	Oscillatory shear index
DNR	Dome-to-neck ratio
PComm	Posterior communicating artery
AComm	Anterior communicating artery
MCA	Middle cerebral artery
ACA	Anterior cerebral artery
ICA	Internal carotid artery
PICA	Posterior inferior cerebellar artery
SCA	Superior cerebellar artery
AICA	Anterior inferior cerebellar artery
MRI	Magnetic resonance imaging
CTA	Computed tomography angiography
MRA	Magnetic resonance angiography
DSA	Digital subtraction angiography
3DRA	3D rotational angiography
MPRAGE	Non-enhanced magnetization-prepared rapid acquisition gradient echo
OCT	Optical coherence tomography
RRT	Relative residence time
CFD	Computational fluid dynamics
2D-DSA	2D digital subtraction angiography
BAC	Balloon-assisted coiling
SAC	Stent-assisted coiling
FD	Flow diverter
PITA	Pipeline Embolization Device for the Intracranial Treatment of Aneurysms
PED	Pipeline Embolization Device
PUFS	Pipeline for Uncoilable or Failed Aneurysms
DMSO	Dimethyl sulfoxide
ISAT	The International Subarachnoid Aneurysm Trial
BRAT	The Barrow Ruptured Aneurysm Trial
